# Anti-anthropomorphism and Its Limits

**DOI:** 10.3389/fpsyg.2018.02205

**Published:** 2018-11-15

**Authors:** Domenica Bruni, Pietro Perconti, Alessio Plebe

**Affiliations:** Department of Cognitive Science, University of Messina, Messina, Italy

**Keywords:** anthropomorphism, common sense, animal rights, ethology, comparative cognition, Morgan's canon

## Abstract

There is a diffuse sentiment that to anthropomorphize is a mild vice that people tend to do easily and pleasingly, but that an adult well educated person should avoid. In this paper it will be provided an elucidation of “anthropomorphism” in the field of common sense knowledge, the issue of animal rights, and about the use of humans as a model in the scientific explanation. It will be argued for a “constructive anthropomorphism,” i.e., the idea that anthropomorphism is a natural attitude to attribute human psychological features to other individuals, no matter they are actually rational agents, or not. If we know the “grammar” of this attitude, we can avoid the risks in overestimatinasg the environmental inputs toward anthropomor-phism and, at the same time, take the heuristic advantages of anthropomor-phism in the use of human mind as a model for both everyday circumstances and scientific enterprise.

## 1. Introduction

There is a diffuse sentiment that to anthropomorphize is a mild vice, nothing really harmful, that people tend to do easily and pleasingly, but that an adult well educated person should avoid. This paper tries to inquire why it is so, and some limits of this latter statement. Note that this question would be ill posed, if anthropomorphism had been a plain logical mistake, as if people when anthropomorphize posit an identity between human nature and the other entity. This seems not the case, in general people who anthropomorphize is well aware that the entity at hand is not identical in nature with a human being, just that certain features, certain overt behaviors, or certain inner mechanisms, are shared. Therefore, there is no fundamental difference between anthropomorphism and common mental practices, like metaphorical thinking, where a selection of features of one domain are ascribed to a different domain. In thinking about a spaceship one assumes similarity between crossing world's open ocean and wandering in the wide space, that implies, for example, being equipped for long term self-sufficency.

It is out of the scope of this paper to review psychological theories of why people tend to anthropomorphize. A review covering theories from Heider's “attribution theory” to Humphrey's “natural psychology” and folk psychology is in Gallup et al. (1997), see also (Urquiza-Haas and Kotrschal, 2015). Rather, our aim is to identify domains in which anti-anthropomorphism is often applied, and to discuss the limits of such condemnation.

There seems to be at least three different domains mostly involved in the negative judgments about anthropomorphism, i.e., common sense knowledge and the role of intuitions in the scientific image of the world (section 2); the issue of animal rights and the anti-specism (section 3); and the use of humans as a model in scientific explanation (section 4). We argue that in all these domains anthropomorphism may work as a natural and frugal heuristics. It is, therefore, matter of elucidate how a kind of “middle way” could work and bring together, on the one side, the idea that anthropomorphism is literally wrong and, on the other side, the fact that it seems to be a natural and productive way of thinking. Arbilly and Lotem (2017) argue for a similar claim by their “constructive anthropomorphism.” With their words: “We believe that the natural tendency of using our human experiences when thinking about animals (i.e., the tendency to anthropomorphize) can actually be harnessed productively to generate hypotheses regarding cognitive mechanisms and their evolution” (p. 2). In a similar way, the rationale of our proposal is to sketch out the main advantages to extend to possibility of this idea to the three areas above mentioned.

## 2. A natural attitude

### 2.1. Common sense knowledge as a two-fold creature

Anthropomorphism is held to be deeply grounded in common sense. Take into consideration how people often turn to mobile phones, cars or their pets. In all these cases we behave as if mobile phones, cars or pets had feelings similar to humans (“My phone is completely exhausted tonight,” or, after running the risk of an accident, “This car really wants to kill me!”). Is there something instructive in this way of thinking so typical of common sense? Or, is it simply a childish attitude? There is a scholarly tradition of suspect against common sense, and often both philosophers and scientists take as the core of their businesses to challenge common sense believes. Sellars (1956), for instance, famously argued for a clash between the “manifest image” and the “scientific image” of the world. Intuitions, however, are often used by philosophers to support their arguments and to refuse the other ones. There is a controversy on this issue in the philosophical community (Cappelen, 2012). But, of course, all depends on what we mean by the phrases “intuition” and “common sense knowledge.”

In this paper we suggest that common sense is a kind of ecological knowledge, which makes people fit in everyday circumstances. It is not only matter of popular beliefs shared with most of the people, but also of taking for granted something similar to the list of truisms in George Moore's *A Defense of Common Sense*: “There exists at present a living human body, which is my body. This body was born at a certain time in the past, and has existed continuously ever since…” (Moore, 1925, p. 65). On the whole, we suggest that common sense knowledge is to be considered as a two-fold creature (Perconti, 2013). It should be articulated into a “deep” and a “superficial” level. While the superficial level consists in judgments and beliefs which are culture-dependent, the deep level is made by implicit procedures grounded in human biology, that is, in motor habits, know how schemata, and bodily imagination. They are cognitive devices which seem to be (at least in part) culture-independent. For instance, the belief that “Berlin is a Central European capital” is part of the superficial level of common sense; and, taking from granted that the sun will shine again tomorrow is typical of the deep level of common sense.

In this perspective, anthropomorphism is considered as an adaptive natural mechanism grounded in the human brain and an instinct in the evolutionary history, which lead people to generate (sometimes illusory) representations that force us into selecting the appropriate data from the exterior world, and sometimes into overestimating the role of the environmental inputs rejecting a psychology of their own. This cognitive mechanism is at the basis of many aspects of the deep level of common sense knowledge, like these which lead us to treat mobile phones, cars, and pets as creatures endowed with feelings and intentions.

Anthropomorphism, in fact, basically is an attitude to attribute human psychological features, more than physical ones, to other entities. It is a psychologically and biologically based attitude to consider individuals as bodies ruled by unobservable forces. No matter if the target entity is really endowed with these features, or not. We can't keep us from using this scheme to give sense to our and others' behaviors. For this reason anthropomorphism is at the basis of pervasive social practices such as religious beliefs, loving pets, and comics.

### 2.2. Anthropomorphic mental triggers

In the environment there are several affordances able to evoke this attitude. Let's call them “anthropomorphic mental triggers,” because they are responsible for the automatic activation of the psychological attitude called “anthropomorphism.” Among the mental triggers which lead human creatures toward anthropomorphism, are included: (1) the predisposition to classify differently living from non-living creatures and to easily recognize emotional and personal characteristic features in biological motion; (2) the inclination to recognize meaningful faces in perceptive configurations; (3) the ability to joint attention with other people and to follow their gaze. Similarly to what happens in the “grammar” of visual perception (especially in the tradition of *Gestaltpsychologie*), in which were traced some of those “triggers” able to switch on the visual automatisms that complement the visual scene in a manner consistent with the way the environment is usually made up, also in the case of the “grammar” of the psychological attribution we should understand how these similar triggers work. When the “anthropomorphic instinct” starts up, triggered by the right affordances in the environment, we are forced to ascribe to that individual the same kind of inner life that everybody experiences in one's own introspection. Regarding the ability to automatically recognize whether a certain movement is living or not, and also to associate them a set of secondary traits, such as sex/gender or emotion, it seems that actually human brain processes specifically and automatically information about the movement of living organisms (Johansson, 1973). These latter, in fact, are endowed with kinematic features the human brain is particularly sensitive to. By isolating kinematic information from other perceptual features, Gunnar Johansson first discovered that humans are not only able to detect whether a certain kind of movement can be attributed to a living thing, but even if that thing is a man or a woman, if he or she is walking fast or slow, and in which mood he or she is. In addition to being processed on a selective basis, the information about the living movement is also processed in a spontaneous way by newborns (Simion et al., 2008). To sum up, it is matter of a skill which can be considered as typical of every human being. Another mental trigger for anthropomorphism is the capacity to spontaneously recognize a face in a given perceptual configuration. For humans, faces are the most significant perceptual configuration in which we can happen to come across. As in the case of recognition of the biological movement, even in face recognition the human brain is able to process such information selectively, automatically and very early in the development (Kanwisher et al., 1997).

Human newborns seem hardwired to social life through the spontaneous interpretation of the thoughts and emotions which are conveyed by faces. Sometimes this spontaneous attitude runs the risk to see more faces than actually there are. Everyone experienced having seen a face when, at a second glance, turned out to be nothing. Sometimes this physiological tendency in humans to overestimate the presence of faces in the world can take a pathological pathway, as in “pareidolia” (Hadjikhani et al., 2009). In a similar way, there are many other “pareidolia-like” cases of overestimating environmental cues, like in the cases of mistaken ascriptions of movements performed by not living organisms, or mistaken meaningful gazes to follow. The experimental results which comes from cognitive neuroscience suggest to consider recognizing faces as something that proceeds along two successive stages. At first, quickly and automatically, brain recognizes a certain perceptual configuration like a face. Then, it associates a set of personal type meanings, such as those linked to the rest of the things that we know about the person we are scrutinizing the face (Haxby et al., 2000). First brain identifiers a face and then, at a second glance, provides with the subjective meaning, making first experience of a “perceptual face,” and then of a face provided with a meaning and a personal story.

The third mental trigger we have to consider is the ability to establish an eye contact with a given individual, and to divide the attention with him and a third part, i.e., gaze following and shared attention. From a developmental point of view, gaze following is prior to shared attention (Carpenter et al., 1998). As a result, in the case of shared attention we have not only a simultaneous act of visual attention, but a more complex joint attention event. Not only two individual acts, but one collective psychological event. With the Joseph Call e Michael Tomasello's words: “Joint attention is not just two individuals looking at the same thing at the same time. Joint attention requires that each of the individuals knows that the other is attending to the same thing as they are attending to; that is what makes it a joint, rather than merely a simultaneous, activity […]. To engage in joint attention, therefore, an individual must at the very least be able to understand that another individual may see or attend to something” (Call and Tomasello, 2005, p. 45). The ability to follow other people gaze and to show interest in what seems to affect other individuals monitoring their attention is very early in typical development (Brooks and Meltzoff, 2002). This ability is essential for the communication development of children and for mentalization. In a word, without joint attention, there would be any intentional state or any language, and therefore ultimately any “society” in the human sense of the term. The above mentioned mental triggers prepare humans in a social direction on the basis of the spontaneous recognition of features which typically belong to a human being. Independently of any cultural encoding, humans are naturally led to consider as a person all individuals who happen to come across, to the condition—indeed quite liberal—that they fulfill the expectation to share attention, to express sense in their faces and to move in a way similar to other people. This natural inclination is toward human beings as well as toward not humans, and it precedes any subsequent cultural symbolization. Sometimes, in fact, we are inclined to treat as a person something which, at a second glance, we are forced to consider otherwise (for example, as an animal or a category of individuals not worthy of social respect). The natural inclination we are talking about is not responsible for the subsequent culture-sensitive judgments. Those judgments, in fact, are not based on the same logic which underlies the functioning of mental triggers.

### 2.3. Humanizing technology

Althought, as we have just appreciated, anthropomorphism is a natural way to give sense to other people's behavior by means of the use of the intentional vocabulary, it is at the basis of scientific practices as well, like humanoid robotics, and developmental robotics (Perconti, 2013). To promote an ecological interchange between humans and robots, in fact, the designer has to take into account what is really able to facilitate a human-robot natural relationship. And, the best candidates for this role are again things like the ability to share other people attention, to follow their gaze, to express sense in their faces and move in a similar way to other organisms. For this, anthropomorphic attitude could inspire the computer scientists, when they are engaged in finding the right computational architecture to allow a humanoid robot to have a fruitful interaction with a real person. This ecological worry should inspire the attempts to humanize both robot's bodies and their minds. (Sandini and Sciutti, 2018, p. 2) stress the difference between “illusorily humanizing robots” and the challenge to make them more “humane”: “A humane robot is a robot considerate of humans, that is, one that maintains a model of humans in order to understand and predict human needs intentions, and limitations, while being transparent, legible, and predictable. The ultimate robot may not be anthropomorphic, but it needs to have at least an anthropomorphic mind” It is interesting to note that the above mentioned attributive mechanisms, i.e., the anthropomorphic mental triggers, are good guides to design both humanoid bodies, endowed with the right mentalization cues, and humane robot minds, endowed with the same cognitive abilities to discover these cues in human overt behavior. The general point here is the possibility to consider anthropomorphism as a natural attitude to attribute human psychological features to other entities in order to give sense to their behavior. It is matter of a fast and frugal heuristics (Gigerenzer, 2007; Gigerenzer et al., 2011), which is actually able in everyday circumstances to easily find a way to categorize what is going on in the environment, like in the case you have suddenly to interpret the behavior of a threatening dog in the streets. Furthermore, anthropomorphism works as a matrix to generate hypotheses on cognitive functions and their evolutionary history (Arbilly and Lotem, 2017). But, finally, we (both as scientists and common people) have to keep out to overestimate the presence of human psychological features into inanimate things and in other species. If not under control, anthropomorphism is, in fact, a danger because it conflicts with the principle of parsimony in psychology and, in general, in the scientific enterprise (Morgan's Canon, Occam's Razor, and so on; see below, section 3). But, if we buy the two-fold image of common sense, as above suggested to do, anthropomorphism appears to be a natural attitude regarding the deep level and both a danger (because it is literally wrong) and an opportunity (in the Arbilly and Lotem's sense) regarding the superficial level. This is exactly the “middle way” above and the reason why our anthropomorphism would be constructive in kind.

## 3. An alleged threat against animal rights

### 3.1. Specism and empathy

There are specific reasons for anthropomorphism aversion related with the animal rights movements, curiously at two opposite ends. Anthropomorphism is seen by some supporters of animal rights as internal to specism, that tends to neglect the genuine features of animal species, conflating them in relation to humans only. On the contrary, enemies of animal rights movements accuse to make use of anthropomorphism in mistakenly ascribing sentiments, like feeling pain, to other animals. For these reasons anti-anthropomorphism is the right theoretical attitude in promoting the animal rights movements.

From a practical point of view, however, things are different. The feeling of empathy toward other animals is often driven by an anthropomorphic stance. This is exactly the reason why human empathy is usually about vertebrates, especially mammals, as they have similar physical features to human ones, like eyes, mouth, and biological motion. Human empathy toward these animals does not depend on any prior scientific knowledge on other animals' psychological skills, but simply on the link between anthropomorphism and empathy. And this latter, even nowadays, is actually the main engine of animal rights movements. Not long time ago, the scientific investigation of the other animals' mental faculties, if they experience pain and suffering, was still impossible. First reports on animal behavior were both anthropomorphic and anthropocentric in kind. Before the nineteenth century, what we knew about animals derived by and large from anecdotal stories. The anthropomorphic style and the use of anecdotes is typical also of Charles Darwin's writings. For example: “Dogs exhibit their affection by desiring to rub against their masters […]. I have also seen dogs licking cats with whom they were friends. This habit probably originated in the females' carefully licking their puppies—the dearest object of their love—for the sake of cleansing them” (Darwin, 1872, p. 118).

Darwin's anecdotal style reflects his convictions on a line of continuity within the world of life, a continuity which also includes mental experiences. While contemporary scientific journals were overwhelmed by studies on mental abilities in other animals, mainstreaming scholars still refuse anthropomorphism. The anthropomorphic attitude was traditionally considered as a “cardinal crime” (Broadhurst, 1963, p. 12) or a “dangerous pit” (Breland and Breland, 1966, p. 3). But, doing so, we ignore the legacy of the *Expression of the Emotions in Man and Animals* (Darwin, 1872). Nowadays, however, Darwin is back and his quite liberal attitude is now grounded in the findings in the field of cognitive ethology (Urquiza-Haas and Kotrschal, 2015). Anthropomorphism fails when we do not have enough information on the biology, the evolutionary history and the ecology of the animal we are interested in. But, in the spirit of Darwin's writings, as well as Jane Goodall and Frans de Waal work, if you are aware of the ethological constraints of what we are saying, you can feel free to use our empathy to make a behavioral prediction and then to evaluate it. If the prediction will be right, then anthropomorphism is a good and fruitful attitude. Otherwise, you are aware the reasons why it does not success.

### 3.2. Anthropomorphism without shame

As we will see in section 4.2, anthropomorphism proved useful in the scientific explanation of what it means to be another animal. We observe that this progress has been beneficial from the standpoint of animal rights too. There are some areas of animal research that have drawn vital lymph from anthropomorphism, including animal learning, animal communication, the human/companion animal bond, and the applied ethology. Let us go into some detail. The field of animal learning received new lymph immediately after the abandonment of the behaviorist paradigm. The use of anthropomorphic projections on animal life has shown that a large part of animal behavior is teleological in kind and that animals are not only aware of their actions, but often able to evaluate the consequences of their actions. Many studies on animal communication achieved significant results only after abandoning the objective analysis of sounds and postures that animals adopted in their context. The scenario changed when the interest of scientists has moved on the message which the animal means, shifting the focus from the overt behavior to the mental life of the animal. Also the applied ethology, i.e., the use of principles and methods of comparative ethology and psychology aimed at modifying animal behavior and creating better environments for their lives, benefited from the use of anthropomorphic projections in scientific explanation.

The anthropomorphic turn in cognitive ethology enabled a concrete improvement of the captive environments. An example of this is the change in the type of housing for great apes that has greatly diminished behaviors such as listlessness, masturbation, and other behavioral abnormalities. Thanks to the empathic projection of our possible responses to captivity, the environments have been enriched with other animals, toys and other sources of stimulation. Anthropomorphism, of course, is especially useful with animals we share our daily lives, such as dogs, with which we shared our social world for over 12,000 years. Pets, in fact, have been accepted as an object of scientific study only in recent decades. For a long time, scientists have not considered farmers, livestock breeders and pet owners as a reliable source of information. They preferred long and costly studies and observations of exotic animals whose *Umwelt*, to quote Jakob von Uexküll (1921), was often unknown. Even in this case we have to consider Darwin as a forerunner of the contemporary way of conducting scientific investigations on animals. In order to develop his ideas on domestication, he included in his works the observations on his pets and many reports of livestock breeders.

Anthropomorphism, moreover, contains more methodological caution than can be believed. With the words of Charles Westley Hume, the founder of the Universities Federation for Animal Welfare: “If I assume that animals have subjective feelings of pain, fear, hunger and the like, and if I am mistaken in doing so, no harm will have been done; but if I assume the contrary, when in fact animals do have such feelings, then I open the way to unlimited cruelties. Animals must have the benefit of the doubt, if indeed there be any doubt.” This kind of observations represents the way “constructive anthropomorphism” could show its advantages at the intersection of cognitive ethology and the movement for the animal rights.

## 4. Humans as a scientific model?

A third case where anti-anthropomorphism is often at home, is in philosophy of science. The idea that by eliminating every human perspectival element science will finally become objective is often found at the beginning of the last century. The dominant attitude at that time was a rejection of explanation in science altogether, as a form of anthropomorphism. The desire and need for finding explanations among humans is natural, but to push the concept of understanding beyond these psychological boundaries was held to be illegitimate. For scholars like Pearson (1911) the scope of science is to provide descriptions, better if in mathematical forms, not to explain anything. Carl Hempel reestablished explanation as the most precious achievement of science, by clarifying that justified explanations, purified from human perspectival elements, are those in the form of nomological deductive schemes. In *The Logic of Functional Analysis*, Hempel (1959) identified in functional analysis the alternative forms of explanation affected by the anthropomorphism virus, and therefore scientifically unacceptable. The negative attitude toward scientific explanation first, and functional explanation later, was a sensible rebellion against the long held idealist view that in order to explain natural phenomena one had to go beyond the limitations of science into some other realm such as metaphysics or theology. Today the majority of scientists and philosophers of science are immune from this temptation. Still, when philosophers of science praise for the search of scientific explanations at wide, often feel the need of a preventive defense against the accuse of anthropomorphism. For example Woodward (2003) in *Making things happen*, in defending his interventionist account of causation, includes a section titled *Nonanthropomorphism*.

While in scientific explanation in general the blame for anthropomorphism is mostly for indirectly invoking supernatural causes and purposes for natural phenomena, there is a domain where anthropomorphizing is more directly under accuse. It is comparative cognition. In this domain the worry about the ascription of human traits to nonhuman animals is today widespread and emphasized. For Wynne (2007) “anthropomorphism […] should have no place in an objective science of comparative psychology, and Blumberg (2007, p. 145) argues that “Along with its fellow travelers—mentalism, introspection, and anecdotalism—anthropomorphism has infected the animal behavior literature.”

### 4.1. Morgan's canon

Like for scientific explanation in general, in comparative cognition too condemnation of anthropomorphism has a long history. Its sharpest and most influential verdict come from the so-called “canon” of Morgan (1894), prescribing that “In no case may we interpret an action as the outcome of the exercise of a higher psychical faculty, if it can be interpreted as the outcome of the exercise of one which stands lower in the psychological scale” (p. 53). Quite like for the case of Pearson's hostility toward explanations in science, Morgan's worries against animals' “higher psychical faculty” were raised in reaction against. In this case the target was the thesis of mental continuity of human and nonhuman organisms strongly held by Charles Darwin and George Romanes. In a famous and provocative passage Darwin (1871, p. 105) argued that “the difference in mind between man and the higher animals, great as it is, is certainly one of degree and not of kind.” As we have already discussed in section 3.1, Darwin made large use of anecdotes about animal behavior, Romanes and other scholars were following his example. Even if Morgan rarely criticized directly Darwin or Romanes, his canon was used as a baton against the use of anecdotes and anthropomorphism in the study of animal behavior. There was an even deeper and older philosophical controversy behind the disagreement between Darwin and Morgan. Descartes (1641) articulated the idea of a sharp separation between human and nonhuman animals that mostly influenced Western culture. He developed an extensive description of organic functions in a purely mechanical manner, shared by humans and other animals, drawing a line between minded and unminded beings. In a perspective today dubbed as *mechanomorphism* (Mitchell et al., 1997), Descartes assumed that nonhuman animals are fully equipped with the mechanics necessary for surviving, but are devoid of mind and consciousness, thus lacking any form of feeling and sentience. The first radical opposition to Descartes was proposed by Hume (1739), advocating cross-specif uniformity in explaining animal behavior. The continuity between human and nonhuman animals was enforced by his empiricist view of the mind, structured by perceptual experiences. Darwin embraced an empiricist view of the mind largely inspired by Hume, while Morgan argued for a discontinuity between humans and animals, departing from the empiricist account of mind and behavior. According to Clatterbuck (2016) this fundamental divergence is the root of their different perspective on biology and on the methodology of animal behavior research.

It has been often remarked that, in fact, the standard application of the canon in comparative cognition has been flawed by its misrepresentation. Thomas (1998, p. 156), in referring that, according to Dewsbury (1984), Morgan's canon is “Perhaps the most quoted statement in the history of comparative psychology,” cannot refrain from adding “that perhaps the most misrepresented statement in the history of comparative psychology is Lloyd Morgan's canon.” The historical misuses of Morgan's canon concern mostly two issues: parsimony and anthropomorphism. The canon has been easily conflated with Occam's razor, advocating the explanation with the fewest assumptions. But Morgan was explicit in warning that the simplicity of an explanation is no criterion of its truth. As an example Morgan (1894, p. 54) cited that “to explain the higher activities of animals as the direct outcome of reason” is simpler “than to explain them as the complex results of mere intelligence or practical sense-experience.” More often than not anthropomorphism can offer the most parsimonious explanation (Sober, 2005). In fact, as revealed by Thomas, Morgan had a very liberal view about anthropomorphism, stating that “First, the psychologist has to reach, through induction, the laws of the mind as revealed to him in his own conscious experience […] Both inductions, subjective and objective, are necessary. Neither can be omitted without renouncing the scientific method” (Morgan, 1903, p. 48–49). An early observation of the failure resulting from an orthodox application of Morgan's canon was given by Hebb (1946, p. 88): “A thoroughgoing attempt to avoid anthropomorphic description in the study of temperament was made over a 2-year period at the Yerkes Laboratories […] All that resulted was an almost endless series of specific acts in which no order or meaning could be found. […] the use of frankly anthropomorphic concepts […] provides an intelligible and practical guide to behavior.” Despite its widespread misuse, Morgan's canon is still taught as a basic part of the comparative psychology curriculum, and still defended especially against the risk of anthropomorphism (Karin-D'Arcy, 2005).

Moreover, the attitude against anthropomorphism derived from Morgan extended well beyond comparative psychology, influencing to a certain extent ethology as well (Boakes, 1984). For sure, it is difficult to conceive Konrad Lorenz obeying scrupulously Morgan's canon when surrounded by his honking geese, or when communicating with his tame raven. In fact, ethologists were the first to see themselves as hampered by the strict compliance with anti-anthropomorphism. Hinde (1982, p. 76) complained that “Fear of the dangers of anthropomorphism has caused ethologists to neglect many interest phenomena.” This rebellion grew during the encounter of ethology with cognition inside comparative cognition, as in the words of Griffin (1992, p. 152): “When one carefully examines such charges of anthropomorphism, it turns out that whatever it is suggested that the animal might do, or think, really is a uniquely human attribute. Such an assumption begs the question being asked because it presupposes a negative answer and is thus literally a confession of prejudgment or prejudice.” Soon this new wave of freedom from the strict adoption of Morgan's canon called for reactions, John Kennedy (1992) devoted an entire volume to the condemnation of anthropomorphism, claiming that (p. 55) “Anthropomorphism must take its slice of the blame for a sort of malaise that has lately afflicted the subject of ethology as a whole.”

### 4.2. Anthropomorphism and “anthropodenial”

Despite Kennedy, in the last two decades the assessment of anthropomorphism in cognitive ethology from a philosophy of science perspective had progressed significantly. A new shared view is that applying anthropomorphism can lead to mistakes, as it would its rejection. However, since Morgan, only one type of error was taking into consideration: that of attributing certain human mental characteristics to a nonhuman animal that lacks it, the error called anthropomorphism. For the opposite error, of mistakenly refusing to attribute human mental states to nonhuman animals that actually do possess them, there is even no a name.

In an extended analysis of applying anthropomorphism in cognitive ethology de Waal (1999) introduced a possible name of this error, as “anthropodenial.” Those to persist in the anthropodenial mistake are called by Keeley (2004) “antianthropomorphites.” A useful metaphor of the error implicit in the systematic denial of human characteristics in nonhuman animal is given by Cartmill (2000) as “anthroporenalism.” It is the (p. 841) “urological version of Morgan's Canon [which] would forbid us to interpret an animal's urine as the outcome of humanlike renal events—if we can find any other way of explaining it.” The fact that no physiologist has never praised against the temptations of anthroporenalism is illuminating about the dose of Cartesian narcissism about our mental life, inherent to anti-anthropomorphism in comparative cognition.

Once established that anthropomorphism can be as misleading as anthropodenial in cognitive ethology, the next question is about possible methodological guidelines that allow to discriminate in advance when and how anthropomorphic attitudes are appropriate. De Waal suggests one discrimination, given by the level of anthropocentrism in anthropomorphism. When the view of the researcher is strongly characterized by the common taxonomy of human mental states and attitudes, easily leads to a form of anthropomorphism that naively attributes human feelings to animals without sufficient information. The opposite is what de Waal calls “animalcentric anthropomorphism,” at work when (p. 264) “rather than being anthropomorphistic from a narrowly human perspective, ethologist mostly interpret behavior within the wider contest of species' habits and natural history.” The concept of “biocentric anthropomorphism” offered by Bekoff (2000) is on the same vein, fostering the adoption of mental features common to humans, without a anthropocentric view. More recently, (Buckner, 2013) introduced a further *anthropo-* lexeme, that of “anthropofabulation,” in defining the kind of anthropomorphism more prone to scientific mistakes which, much like for de Waal, is imbued by anthropocentrism. His term is due to the “confabulation about our own prowess” (p. 185) when studying nonhuman animal cognition.

A different answer to the quest for a methodological principle for a correct application of anthropomorphic hypothesis is given by Fitzpatrick (2008) as “evidentialism” (p. 242): “in no case should we endorse an explanation of animal behavior in terms of cognitive process X on the basis of the available evidence if that evidence gives us no reason to prefer it to an alternative explanation in terms of a different cognitive process Y—whether this be lower or higher on the ‘psychical scale’.” The principle has the merit to break the prejudicial asymmetry with respect to the two errors, that of mistaken anthropomorphism and that of mistaken anthropodenial, however is too general for being an effective methodological prescription. This is, instead, the aim of the “critical anthropomorphism” proposed by Gordon Burghardt (1991, 2007). The concept is derived from that of “critical realism” (Mandelbaum, 1964), and is the idea of adopting anthropomorphism in order to generate ideas that may prove useful in planning experiments and gaining understanding in the realm of animal cognition, with the awareness of the risk of drawing anthropomorphic conclusions that are erroneous. The “critical” component is applied by using other sources of information, such as natural history, physiological and neurological constraints, careful behavior descriptions, optimization models, and so forth. More precisely, when exercising critical anthropomorphism care should be applied in avoiding “anthropomorphism by omission,” that is the failure to consider that other animals have a different world than ours (Rivas and Burghardt, 2002). A step further is taken by Timberlake (1997, 2007) by moving from anthropomorphism toward “theromorphism.” This approach involves posing possible complex and human-like cognitive capacities in animals, but adopting an animal-centered view, in his own words Timberlake (2007, p. 142): “A theromorphic approach attempts to discover and represent important aspects of an animal's sensory and motivational worlds, thus allowing a human experimenter/observer to enter the animal's world.”

The attempt to “enter the animal's world” is undoubtedly praiseworthy, but it is an effort intrinsically limited by our human cognitive status. This is but one reason for searching help in anthropomorphism. Probably one of the most viable strategy is to acknowledge that how humans cognition works is necessarily the best known model, therefore to make use of it just as a model. This is the sense of what Arbilly and Lotem (2017) calls “constructive anthropomorphism,” and adopting the human model to animals may provide several advantages, in that (p. 2) “it forces us to consider complex cognitive abilities that are normally not attributed to animals, explain them using simple biological principles, and then, to carefully examine their possible application to animals.”

Let us conclude by illustrating few cases in animal studies, where a “careful” application of anthropomorphism has lead to important discoveries.

von Frisch (1927) identified the famous “dance” performed by honey bees, interpreted as a communication code for informing hive-mates about the location of food just found. Von Frisch's discovery was hardly attacked for ascribing human-like communication abilities to insects, commonly deemed with very limited cognitive capacity. Critics of von Frisch later endorsed several alternative and less cognitively sophisticated explanations. One is that bees are simply conditioned to monitor and follow the odors emitted by returning foragers, with dancing just an irrelevant and unintended artifact with no communicate role (Wenner, 1998). In spite of that resistance, recent investigations have yielded new evidence of the complexity and flexibility of the honey bees dance (Gould and Grant-Gould, 1995; Seeley, 2003). Subtle variations in the way of indicating a direction distinguish between reporting locations of possible new hive sites or locations of food sources. Moreover, there can be comparisons between proposals of different locations, with bees first dancing signaling the source they found then following dancers describing a different source, and finally dance about the latter. In this case the term “dance” itself is just metaphor of a human behavior, but anthropomorphism is applied in hypothesizing a complex form of communication, and is applied as “animalcentric anthropomorphism,” in that the communication medium and the aims of communicating are cast from the world view of the animal.

Unlike “dancing” for von Frisch, when Panksepp (1998) first wrote about “laughing” rats, he did use this verb with its literal meaning, for a behavior that is considered uniquely human. He noted a regular chirp in rats, with a trill type modulation around a frequency of 50kHz, seemingly related with positive emotional states. The following decades of research have yielded wide evidence supporting this bluntly anthropomorphic claim (Panksepp and Burgdorf, 2003; Burgdorf et al., 2005; Burgdorf and Panksepp, 2006). Not only do rats chirp when aroused to playful activities, paralleling children's playful laughter, they do the same also during tickling. Further, the same behavior can be elicited with electrical stimulation of the brain, on neural circuits involved in positive emotional responses, shared by most mammals, humans included.

Attributing laughing to rats is certainly not within the common sense anthropomorphism repertoire. As an opposite end we found “play,” that is probably one of the preferred attribution of human habits to other animals by lay people, especially those keen on animals. This is one of the reasons that marginalized the scientific study of animal play: “having fun” is a too distinctively human feature. Niko Tinbergen (1963, p. 413) argued that play was too biased by “subjectivist, anthropomorphic undertones” to be seriously studied. By releasing anti-anthropomorphism worries, ethology progressed greatly the science of play, human playing included (Panksepp, 1981; Waring, 1983; Bekoff, 1984, 2001; Burghardt, 2005). Curiously, a recent line of research uses dog-human play as a testbed for studying anthropomorphism itself using ethological methods (Horowitz and Bekoff, 2007). The methodology is to classify behaviors by dogs in play and to compare with the behavior of projective anthropomorphizing by humans playing with them.

## 5. Conclusions

Anthropomorphism is, of course, literally wrong. But, it is also a natural cognitive attitude, grounded in the human biology and consisting in many natural inclinations that lead human beings to consider a certain individual in the world as a person, no matter if that individual actually is a rational agent, or not. As above argued for, anthropomorphism can be considered as a frugal heuristics both for everyday life and the scientific explanation. The constructive side of anthropomorphism is a major component in understanding that common sense knowledge plays an ecological role in everyday knowledge. Moreover, understanding the “physiology” of the use of anthropomorphism, and its advantages, also allows us to avoid the risks of its “pathology,” hidden in overestimating those environmental cues, which are able to elicit the logical pathways which lead humans to see too much “human” in the world.

## Author contributions

Although all authors contributed equally to this work, DB worked mostly on section An Alleged Threat Against Animal Rights, PP mostly on section A Natural Attitude, and AP mostly on section Humans as a Scientific Model?

### Conflict of interest statement

The authors declare that the research was conducted in the absence of any commercial or financial relationships that could be construed as a potential conflict of interest.
